# Evaluation of the appropriate use of chest CT-Scans in the diagnosis of hospitalized patients in shiraz teaching hospitals, Southern Iran

**DOI:** 10.1186/s12962-022-00381-0

**Published:** 2022-08-23

**Authors:** Ramiz Kamrani, Mohammad Javad Fallahi, Seyed Masoom Masoompour, Seiyed Mohammad Ali Ghayumi, Reza Jalli, Sepideh Khederzadeh, Amirhossein Erfani

**Affiliations:** 1 Department of Internal Medicine, Shiraz University of Medical Sciences, Shiraz, Iran; 2 Non-Communicable Diseases Research Center, Shiraz University of Medical Sciences, Shiraz, Iran; 3 Medical Imaging Research Center, Shiraz University of Medical Sciences, Shiraz, Iran; 4 Thoracic and Vascular Surgery Research Center, Shiraz University of Medical Science, Shiraz, Iran; 5 Department of Internal Medicine, Namazi Hospital, Namazi Square, Shiraz, Fars Iran

**Keywords:** Computed tomography, Thorax, Pulmonologist, Radiology, Radiography

## Abstract

**Purpose:**

During recent years, overuse of medical imaging especially computed tomography has become a serious concern. We evaluated the suitable usage of chest computed tomography (CT)-scan, in patients hospitalized in emergency and medical wards of two teaching hospitals of Shiraz University of Medical Science.

**Methods:**

Medical records of 216 patients admitted in two major teaching hospitals (Namazi and Shahid Faghihi), who had undergone chest radiography and at least one type of chest CT were investigated. The clinical and paraclinical manifestations were independently presented to three pulmonologists and their opinion regarding the necessity and type of CT prescription were documented. Also, the patient’s history was presented to an expert chest radiologist and asked to rate the appropriateness of chest CT according to American colleague of radiologist (ACR) criteria.

**Results:**

In 127 cases (59%), at least 2 out of 3 pulmonologists had the same opinion on the necessity of performing CT scan regardless of CT scan type, in 89 cases (41%) the same CT type and in 38 (17.5%) cases other CT type was supposed. Based on ACR criteria, of total prescribed CTs, 49.5% were “usually not appropriate” and 31.5% of cases were “usually appropriate”. Among 109 pulmonary CT angiography, 54 (49.5%) was usually not appropriate base on ACR criteria, which was the most frequent inappropriate requested CT type.

**Conclusion:**

Considering the high rates of inappropriate utilization of chest CT scan in our teaching hospitals, implementation of the standard guideline at a different level and consulting with a pulmonologist, may prevent unnecessary chest CTs prescription and reduce harm to patients and the health system.

## Introduction

Over the past three decades, computed tomography (CT) scans have significantly been used in routine practice; more than 70 million CT scans were reported in the United States in 2007 [1]. Technology has recently changed the basis for the diagnoses and management of common chest diseases. The chest CT scans are valuable tools to examine abnormalities on radiographs including; single pulmonary nodule, masses of the mediastinum, lung cancer classification, and diagnosis of pulmonary metastases, and aortic diseases. Even, during the current novel coronavirus disease 2019 (COVID-19) pandemic, the vital role of low dose chest CT in diagnosis and severity classification of severe acute respiratory syndrome coronavirus 2 (SARS-CoV-2) infection has been documented, especially for differentiating from other similar entities [2–4].

Although CT scan provides valuable information as an integral part of the diagnosis, the overuse of CT scan has become a serious concern. Recently, the CT scan overuse has been debated in scientific guidelines; Since it is accompanied by adverse radiation effects, unpredictable reactions to contrast, and economic burden. Furthermore, due to limited resources, this will deprive patients who are in more need of these services [5].

Overutilization was described when imaging interventions do not alter the patient's treatment process or the outcome of treatment. Studies have shown that between 20–35% of chest CT imaging may be unnecessary [6, 7]. Finding the proper balance between the use of ever-increasing medical imaging, along with its costs and disadvantages, is something that health policymakers face.

Due to the need for planning proper imaging interventions, and the lack of indigenous studies in our country; In this study, based on the clinical suspicion of pulmonologists and criteria of the American college of radiology (ACR) [8], the appropriate use of chest CT scan in patients admitted to the emergency and medical wards of two major teaching hospitals (Namazi and Shahid Faghihi) affiliated to the Shiraz University of medical sciences were examined.

## Methods

The present study was designed and conducted as a prospective cross-sectional study. Medical records of 216 adult patients admitted in two major teaching hospitals (Namazi and Shahid Faghihi) affiliated to Shiraz University of medical sciences were included from the first of November 2018 to the end of February 2019. Simple random sampling was done over 4 months among all hospitalized patients who had a chest x-ray, as well as a type of chest CT scan. The admitted patients in emergency and medical wards were managed by their treating physicians (resident of internal medicine, general internist, or subspecialists of internal medicine).

Data were collected using a questionnaire that included: name, age, sex, job, previous hospital admission for similar reasons, initial chief complaint, vital signs at the time of admission, medical and social history, and clinical examination findings. The patients who were discharged at the time of completing the questionnaire were excluded from the study. The study protocol was approved by the Medical Ethics Committee and the Research Vice-Chancellor of Shiraz University of Medical Sciences, and verbal informed consent was obtained from all patients.

We followed two methods to determine the chest CT appropriateness; first through an expert panel of three pulmonologists, and second via comparison with the American college of radiology (ACR) by an expert chest radiologist.

The collected data were presented to three pulmonologists (MJF, SMM, SMG), who had practiced pulmonary medicine for at least 10 years. The pulmonologists evaluated the initial patients' data and their plain chest radiograph independently and were blinded from the requested CT scan which was performed for the patient; then decided if the patient needs to undergo a chest CT scan or not. If the patient needs to undergo chest CT, which type of CT scan including high resolution, spiral without contrast, spiral with contrast, or pulmonary angiographic CT scan is indicated. If at least two pulmonologists agreed with the CT scan and its type, the CT scan deems appropriate. To determine the appropriateness of performed chest CT scans based on the American college of radiology (ACR), an expert chest radiologist (R.J) evaluated and qualified their appropriateness in comparison to ACR scenarios [8].

The effective radiation dose of chest CT protocols based on our study instruments included: PTE protocol: 6.16 mSv, spiral chest CT with contrast:3 mSv, chest HRCT can:2.114 mSv, chest CT without contrast:2 mSv.

Considering the confidence level of 95%, the margin of error of 6%, and the estimated prevalence of inappropriate chest CT request of 25%, the minimum sample size would be 200. The variables are reported as mean ± standard deviation (SD) or frequency and percentile. The Kappa coefficient was calculated and reported for agreement between pulmonologist opinion and ACR criteria.

## Results

Among 216 patients, 107(49.5%) of participants were female. The mean age of participants was 57.0 ± 19.5 years and 100 (46%) was smoker, 124 (57.4%) admitted in the emergency ward.

The most common chief complaint was shortness of breath (39.4%), cough associated with shortness of breath (25.5%). Other clinical manifestations and comorbidities are summarized in Table 1.

**Table 1:** Frequency of demographic and clinical features among investigated patients

Variable	No. (%) or mean ± SD
Age	57.0 ± 19.5
Gender	
Male	109 (50.5)
Female	107 (49.5)
Marital Status	
Single	43 (19.9)
Married	173 (80.1)
Educational level	
Under-diploma	96 (44.4)
Diploma	(68 (31.5)
Bachelor’s degree	46 (21.3)
Master’s degree	6 (2.8)
Social History	
non-smoker, non-baker	116 (53.7)
Smoker, ex-smoker	87 (40.3)
Baker	13 (6.0)
Admission ward	
Emergency	124 (57.4)
Internal medicine	47 (21.8)
Intensive care unit	14 (6.5)
Pulmonary ward	14 (6.5)
Other	17 (7.9)
Chief complaints	
Dyspnea	85 (39.4%)
Cough and dyspnea	55 (25.5%)
Hemoptysis	22 (10.2%)
Generalized weakness	19 (8.8%)
Loss of consciousness	16 (7.4%)
Fever	10 (4.6%)
Others	9 (4.2%)
Comorbidity(patient’s history)	
Hypertension	59 (27.3%)
Diabetes Mellitus	45 (20.8%)
Obstructive lung disease	35 (16.2%)
Malignancy	33 (15.3%)
Ischemic heart disease	30 (14%)
Restrictive Lung disease	4 (1.9%)
Vital Signs on admission	
Systolic BP (mmHg)	119.6 ± 19.7
Diastolic BP (mmHg)	75.6 ± 10.0
Pulse rate/minute	96.6 ± 14.9
Respiratory rate/minute	19.1 ± 3.3
Temperature (Ċ)	37.3 ± 0.5
O_2_ Saturation(%) on room air	89.7 ± 7.0
O_2_ Saturation(%) with support	91.6 ± 9.8
Lung auscultation	
Normal	44 (20.4)
Rales	81 (37.5)
Decreased sound	43 (19.9)
Rhonchi	34 (15.7)
Wheezing	8 (3.7)
Rales and wheezing	6 (2.8)
Physical examination	
Prominent JVP	39 (18.1)
Lymphadenopathy	13 (6.0)
Cyanosis	11 (5.1)
Peripheral edema	64 (29.6)
Chest deformity	1 (0.5)
Radiography in past 3 months	
Yes	37 (17.1)
History of admission due to similar complaint	
Yes	92 (42.6)

*JVP* jugular venous pressure *BP* blood pressure, *SD* Standard deviation

Among 216 CT scans of chests, 56, 100, and 60 cases were requested by medical residents, internist, and medical subspecialties respectively. The most common type of CT requested by physicians was pulmonary CT angiography (109: 50.5%), followed by spiral chest CT with contrast (65: 30.1%) (Table 2).

**Table 2:** Frequency (%) of primary and final diagnosis among the investigated population

Disease	Primary diagnosis	Final diagnosis
Pulmonary thromboembolism	45 (20.8)	28 (13.0)
Pneumonia	38 (17.6)	34 (15.7)
Miscellaneous	29 (13.4)	45 (20.8)
Heart failure or pulmonary edema	22 (10.2)	18 (8.3)
malignancy (primary/ secondary)	21 (9.7)	27 (12.5)
Chronic obstructive pulmonary disease	18 (8.3)	16 (7.4)
Pleural effusion	14 (6.5)	13 (6.0)
Interstitial lung disease	12 (5.6)	11 (5.1)
Tuberculosis	10 (4.6)	14 (6.5)
Bronchiectasis	4 (1.9)	7 (3.2)
Aspiration pneumonia	3 (1.4)	3 (1.4)

Pneumonia (15.7%), pulmonary embolism (13%), and lung mass or metastasis (12.5%) were the most common causes of hospitalization among patients. The frequency of primary and final diagnosis among the investigated population was illustrated in Table 2.

### Appropriateness regarding pulmonologist view

At least 2 out of 3 pulmonologists in 127 cases (59%) had the same opinion on the necessity of performing CT scan regardless of CT scan type, in 89 cases (41%) the same CT type and in 38(17.5%) cases other CT type was supposed. In 82 cases (38%) pulmonologists agreed that CT scans were not indicated. In seven cases (3%), there was no agreement between pulmonologists in the necessity of CT scan or its type.

The most common type of chest imaging required by pulmonologists opinion was spiral chest CT with contrast (23.1%) followed by pulmonary CT angiography (20.8%). According to this result, there was a moderate agreement between three pulmonologists in CT appropriateness (kappa:0.38, and p value < 0.0001). Table 3 shows frequency (percent) of CT-scan types requested by physician and CT scans which were recommended by the pulmonologists.

**Table 3:** Frequency (%) of CT-scan types requested by the physician based on pulmonologists recommendation

Requested by physician	Pulmonologists' recommendation n (%)					
Type of imaging	Pulmonary CT angiography	Spiral with contrast	HRCT	CT without contrast	CT is not indicated	No agreements
Pulmonary CT angiography (109)	**43 (39.4%)**	17 (15.6%)	5 (4.6%)	0 (0%)	40 (36.7%)	4 (3.7%)
Spiral with contrast (65)	1 (1.5%)	**30 (46.2%)**	9 (13.8%)	0 (0%)	22 (33.8%)	3 (4.6%)
HRCT (29)	0 (0%)	1 (3.4%)	**13 (44.8%)**	0 (0%)	15 (51.7%)	0 (0%)
CT without contrast (13)	1 (7.7%)	2 (15.4%)	2 (15.4%)	**3 (23.1%)**	5 (38.5%)	0 (0%)
Total (216)	45 (20.8%)	50 (23.1%)	29 (13.4%)	3 (1.4%)	82 (38%)	7 (3.2%)

*CT* Computed tomography, *HRCT* High-resolution computed tomography; bold number indicate concordance between patient’s physician and at least 2/3 pulmonologist

### Appropriateness regarding ACR criteria

According to the ACR criteria, 49.5% and 12.5% of CT scans were usually appropriate and may be appropriate, respectively. 6.5% of patients compliant were not defined in ACR criteria (Table 4).

**Table 4:** Frequency (%) of chest CT type and their appropriateness based on ACR appropriateness criteria

Chest CT type	Frequency	ACR criteria appropriateness			
		Usually appropriate	Maybe appropriate	Usually not appropriate	No definition
Pulmonary CT angiography	109	48 (44%)	2 (1.8%)	54 (49.5%)	5 (4.6%)
Spiral chest CT with contrast	65	34 (52%)	8 (12.3%)	8 (12.3%)	6 (9.2)
Chest HRCT	29	16 (55.2%)	7 (24.1%)	3 (10.3%)	3 (10.3%)
Chest CT without contrast	13	9 (69.2)	1 (7.7)	3 (23.1%)	0 (0%)

*CT* computed tomography, *HRCT* High-resolution computed tomography

The type of CT application was more appropriate in cases with hemoptysis or shortness of breath, and in other complaints such as fever and decreased level of consciousness, the number of inappropriate requests was higher (Table 5).

**Table 5:** CT-scan requisition appropriateness by chief complaint and requesting physician based on ACR criteria

Variable	Frequency; n (%)	ACR criteria			
		Usually appropriate	Maybe appropriate	Usually Not appropriate	No definition
Chief complaint					
* Hemoptysis*	22 (10)	15 (68.2)	2 (9.1)	4 (18.2)	1 (4.5)
* Dyspnea*	85 (39)	49 (57.6)	8 (9.4)	23 (27.1)	5 (5.9)
* Cough and dyspnea*	55 (25)	26 (47.3)	7 (12.7)	20 (36.4)	2 (3.6)
* Loss of consciousness*	16 (7)	3 (18.8)	2 (12.5)	10 (62.5)	1 (6.3)
* Generalized weakness*	19 (9)	9 (47.4)	6 (31.6)	1 (5.3)	3(15.8)
* Fever*	10 (5)	3 (30)	2 (20)	4 (40)	1 (10)
* Others*	9 (5)	2 (22.2)	0 (0)	6 (66.7)	1 (11.1)
* Total*	216 (100)	107 (49.5)	27 (12.5)	68 (31%)	14 (6.5)
Imaging requested by					
* Resident*	56 (25.9)	34 (60.7)	3 (5.4)	19 (33.9)	0 (0)
* Specialist*	100 (46.3)	42 (42)	10 (10)	39 (39)	9 (9)
* Subspecialist*	60 (27.8)	31 (51.7)	14 (23.3)	10 (16.7)	5 (8.3)
* Total*	216 (100)	107 (49.5)	27 (12.5)	68 (31.5)	14 (6.5)

Among CT scans that were requested by the medical resident, internist, and medical subspecialties, 60.7%, 42%, and 51.7% were usually appropriate, respectively (Table 5).

**Fig. 1:**
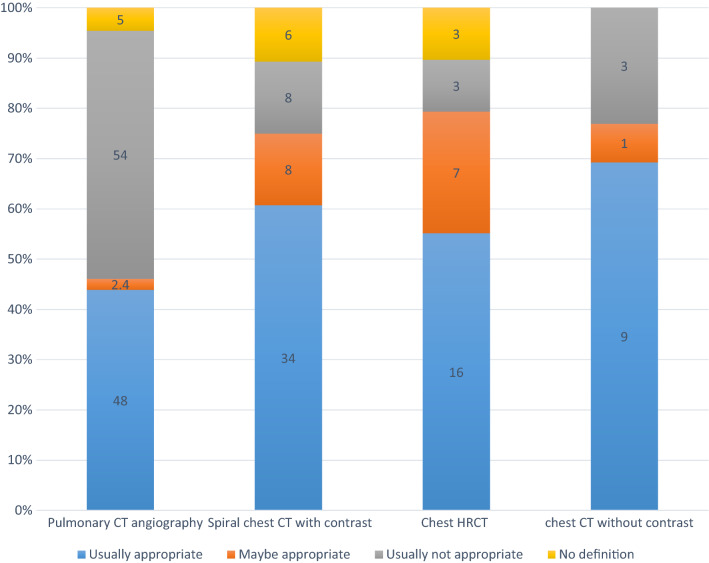
Frequency (%) of chest CT type and their appropriateness based on ACR appropriateness criteria. *CT* computed tomography, *HRCT* High-resolution computed tomography

Among 109 pulmonary CT angiography, 54 (49.5%) was usually not appropriate base on ACR criteria, which was the most inappropriate requested CT type. The frequency and appropriateness of other CT types are summarized in Fig. 1.

There was a fair agreement between pulmonologist and ACR criteria (Kappa = 0.227 with p < 0.0001) when the agreement was defining at least 2/3 pulmonologist opinion and “usually appropriate” and “maybe appropriate” ACR classification. From 127 CT scans (regardless of type) which were indicated by pulmonologists' decision, 110 (86.6%) was also appropriate by ACR criteria. From 82 CT scans that were not indicated by pulmonologists' decision, 49 (60%) CT scans were not appropriate by ACR criteria.

Among the 82 (38%) of the cases in which the pulmonologists agreed that CT was not indicated, the majority were regarding PTE protocols (40; 48.8%), followed by spiral chest CT with contrast (22; 26.8%), HRCT (15; 18.3%), and spiral CT scans (5; 6.1%). Also, the most frequent diagnosis of these patients was heart failure or overload (16; 19.5%), pneumonia (13; 15.9%), and COPD (11; 13.4%), in which the imaging approved the preliminary diagnosis in 72 (87.8%) of the cases and in 10 (12%) patients, the final diagnosis was changed. Based on ACR criteria, only 21 (25.6%) of these patients had indications, however, in the majority of cases (72.87.8%), the pulmonologists reported that imaging was extra and unnecessary work-up. Also, 16 (19.5%) of these patients had a recent imaging in the past 3 months.

A more detailed analysis regarding the appropriateness of CT use based on ACR criteria among the variables in our study is demonstrated in Table 6.

**Table 6:** Evaluation of indication of computed tomography scan based on American College of Radiology (ACR) criteria. (N = 202)

Variable	ACR Criteria		P-value
	Indicated; n = 134	Not-indicated; n = 68	
Age (years); mean ± SD	56.4 ± 19.1	57.8 ± 20.4	0.617
Gender; n (%)			
Male	70 (68.6)	32 (31.4)	0.487
Female	64 (64.0)	36 (36.0)	
Marital Status; n (%)			
Single	29 (72.5)	11 (27.5)	0.357
Married	105 (64.8)	57 (35.2)	
Educational level; n (%)			
Under-diploma	57 (63.3)	33 (36.7)	0.312
Diploma	43 (69.4)	19 (30.6)	
Bachelor's degree	28 (63.6)	16 (36.4)	
Master's degree	6 (100)	0 (0)	
Social History; n (%)			
Negative	63 (61.2)	40 (38.8)	0.201
Smoker	63 (73.3)	23 (26.7)	
Baker	8 (61.5)	5 (38.5)	
Admission ward; n (%)			
Emergency	78 (67.8)	37 (32.2)	0.653
Non-Emergency	58 (64.4)	31 (35.6)	
Chief complaints; n (%)			
Dyspnea	57 (71.3)	23 (28.7)	**0.001**
Cough and dyspnea	33 (62.3)	20 (37.7)	
Hemoptysis	17 (81.0)	4 (19.0)	
Generalized weakness	15 (93.8)	1 (6.3)	
Loss of consciousness	5 (33.3)	10 (66.7)	
Fever	5 (55.6)	4 (44.4)	
Others	2 (25.0)	6 (75.0)	
Comorbidity; n (%)			
Hypertension	35 (64.8)	19 (35.2)	0.782
Diabetes Mellitus	26 (60.5)	17 (39.5)	0.358
Obstructive lung disease	18 (66.7)	9 (33.3)	0.969
Malignancy	17 (68.0)	8 (32.0)	1.000
Ischemic heart disease	15 (57.7)	11 (42.3)	0.308
Restrictive Lung disease	2 (66.7)	1 (33.3)	1.000
Vital Signs on admission; mean ± SD			
Systolic blood Pressure	119.7 ± 17.9	119.9 ± 23.2	0.963
Diastolic blood Pressure	75.9 ± 8.9	75.7 ± 12.2	0.913
Pulse rate	96.8 ± 13.9	95.6 ± 15.9	0.610
Respiratory rate	19.1 ± 3.4	19.3 ± 3.1	0.644
Temperature	37.2 ± 0.5	37.3 ± 0.6	0.289
O_2_ Saturation without support	89.8 ± 6.9	89.7 ± 7.3	0.869
O_2_ Saturation with support	90.8 ± 12.3	92.7 ± 2.5	0.394
Lung auscultation; n (%)			
Normal	25 (61.0)	16 (39.0)	0.416
Abnormal	109 (67.7)	52 (32.3)	
Physical examination; n (%)			
Prominent JVP	19 (50.0)	19 (50.0)	**0.018**
Lymphadenopathy	7 (70.0)	3 (30.0)	0.801
Cyanosis	7 (70.0)	3 (30.0)	0.801
Edema	32 (56.1)	25 (43.9)	0.055
Chest deformity	0 (0)	1 (100)	0.335
Radiography in past 3 months; n (%)	23 (71.9)	9 (28.1)	0.545
History of admission due to similar complaint; n (%)	83 (69.7)	36 (30.3)	0.230
Disease Final diagnosis; n (%)			
Pulmonary thromboembolism	24 (88.9)	3 (11.1)	**0.008**
Pneumonia	21 (61.8)	13 (38.2)	0.536
Miscellaneous	28 (68.3)	13 (31.7)	0.854
Heart failure or overload	3 (17.6)	14 (82.4)	** < 0.001**
malignancy (primary/ secondary)	21 (84.0)	4 (16.0)	**0.046**
Chronic obstructive pulmonary disease	7 (46.7)	8 (53.3)	0.094
Pleural effusion	6 (54.5)	5 (45.5)	0.513
Interstitial lung disease	8 (80.0)	2 (20.0)	0.500
Tuberculosis	10 (83.3)	2 (16.7)	0.344
Bronchiectasis	5 (71.4)	2 (28.6)	1.000
Aspiration pneumonia	1 (33.3)	2 (66.7)	0.263

*ACR* american college of radiology, *JVP* jugular venous pressure, LAP: *SD* Standard deviation

Bold variables indicate a significant association

As demonstrated in Table 6, among the variables in our study, higher rates of CT scan without indication were observed among patients with chief complaint of loss of consciousness and patients with a diagnosis of heart failure or overload. Also, appropriate CT scans were significantly higher among patients with malignancies and PTE.

## Discussion

In our study, based on a pulmonologist's view, 59% of chest CT (regardless of type) was truly indicated. Also, based on ACR criteria, collectively, 62% of requested CT scan was “usually appropriate” and “maybe appropriate”.

The various trend of CT imaging utilization was recently evaluated [9–12] but few studies focus on its appropriateness. Appropriate use of medical imaging was surveyed in two Spanish hospitals which revealed that almost half of imaging was inappropriate which is somewhat similar to our result [6]. Previous studies by Cristofaro et al. and Moriarity et al. suggested that between 26 and 44 percent of CT scans and Magnetic resonance imaging (MRI) may be inappropriate for the patient's diagnosis, given the ACR guideline [13, 14]. Lehnert et al. analyzed the appropriateness of 459 outpatients CT and MRI examination of two academic medical center radiology benefit management company and found that 26% was inappropriate. About 12% of chest CT and 30% of the chest and abdomen and pelvic CT scan was inappropriate [15]. Another study evaluated the appropriate use of CT and MRI in British Columbia and a very low rate of inappropriateness (2%) was documented [16]. Several reasons explain the heterogeneous finding of the above studies. Among them, are the type of evaluated imaging (MRI, CT), body site of imaging, the setting of imaging (outpatient vs inpatient, emergency vs inpatient ward), measurement tool (ACR criteria, expert opinion, local guideline, and insurance company guideline).

In line with Bianco et al.'s study, the overall appropriateness regarding the ACR criteria for hemoptysis in our study was high [7]. We found that Chest CT in the context of loss of consciousness was not appropriate in the majority of patients since the primary cause of decreased consciousness is mostly due to CNS disease, acute on chronic respiratory failure, and drug overdose, in which chest CT is not useful in a majority of these patients.

Assessing the appropriateness of the type of CT required by the physician based on the ACR criteria indicates that in most cases the type of CT prescribed has not been appropriate for the patient. various chest CT protocol have different effective radiation dose (PTE protocol: 6.16 mSv, spiral chest CT with contrast:3 mSv, chest HRCT can:2.114 mSv, chest CT without contrast:2 mSv based on our CT machine). besides ionizing radiation dose, requisite of contrast and their acute and long-term adverse effects, the diagnostic yield of various chest computed tomography protocol differ for different diagnosis. Among the different types of CT performed for patients in our study, the pulmonary thromboembolism (PTE) protocol was the most common type of CT, but according to the ACR standard criteria, in 49.5% of cases, they were usually inappropriate. However, this diagnosis has been incorrect based on ACR criteria and by pulmonologists' opinion. This degree of inappropriate chest CT evaluation for pulmonary embolism was shown by Hutchinson et al. [17] which showed that only 19% of chest CT with pulmonary emboli protocol ultimately confirmed diagnosis. Some measures such as higher d- dimer ordering and formal thromboembolism risk factor assessment were hypothesized to decrease inappropriate imaging utilization for pulmonary thromboembolism [18, 19]. Studies have shown that a quality improvement program [20–23] and a single educational program do not change this behavior [24]. Defensive practice and pressure to increase emergency department turnover were supposed as a barrier to decrease CT utilization for pulmonary embolism [20] which highly apply to our crowded emergency ward.

Based on ACR criteria; internists (specialists) in charge of the emergency ward had more inappropriate CT requests (42%). These results may be since internists must visit a high number of patients in emergency wards and therefore have less time for evaluation and decision, leading to prescribing more diagnostic Para clinic tools because of legal issues to reduce misdiagnosis and mismanagement. On the other hand, more time and experience of subspecialists play a role in less inappropriate CT requests. integrating point-of-care clinical decision support based on ACR criteria or local guidelines, the implementation of a provider-led radiology medical management program may improve the appropriateness of imaging requests especially by an internist or medical resident [14, 25].

The results showed that the opinion of pulmonologists was in fair agreement with the ACR criteria. So, we propose that in addition to the implementation of ACR criteria at the level of the physician in charge and radiologist, an expert pulmonologist consultation even by phone call may decrease inappropriate CT request to some extent.

Our study had some limitations. we did not evaluate those patients who not undergone a chest CT scan. Some of these patients may truly benefit from advanced chest imaging and including this population may change the degree of appropriateness. Also, our pulmonologist opinions were based on recorded patient data and admission chest X-ray. It is possible that their decision was different and more concordant with ACR criteria if they visit patients in person. It should be noted that no decision-making system is complete and free of fault, as we have shown that 10 out 82 patients who should not be undergone chest CT imaging based on pulmonologist opinion, the performed chest CT had changed the final diagnosis.

## Conclusion

The present study found that a considerable portion of requested chest CT was either not indicated or should be done with a different protocol. Therefore, in general, and in comparison, with the statistics of other regions of the world, it can be said that the status of CT prescribing in at least two educational hospitals of Shiraz University of Medical Sciences is somewhat similar to the results of other studies, and subsequently needs improvement. One issue that could lead to this upgrade process is the emphasis on standard guidelines that, if properly trained and implemented, along involving pulmonologist decision making, can benefit the patient, the treatment system, and health care, and prevent unnecessary costs and ensure the patient's present and future health.

## Data Availability

All data generated or analyzed during this study are included in this manuscript. Please write to the corresponding author for further information.

## Abbreviations

ACR: American colleague of radiologist
CT: Computed tomography
COVID-19: Coronavirus disease 2019
MRI: Magnetic resonance imaging
PTE: Pulmonary thromboembolism
SARS-CoV-2: Severe acute respiratory syndrome coronavirus 2
SD: Standard deviation
